# Model-Guided Metabolic Rewiring for Gamma-Aminobutyric Acid and Butyrolactam Biosynthesis in *Corynebacterium* *glutamicum* ATCC13032

**DOI:** 10.3390/biology11060846

**Published:** 2022-05-31

**Authors:** Yun Zhang, Jing Zhao, Xueliang Wang, Yuan Tang, Shuwen Liu, Tingyi Wen

**Affiliations:** 1 CAS Key Laboratory of Pathogenic Microbiology and Immunology, Institute of Microbiology, Chinese Academy of Sciences, Beijing 100101, China; zj19951009@163.com (J.Z.); wangxueliang18@mails.ucas.edu.cn (X.W.); tangyuan20@mails.ucas.ac.cn (Y.T.); liusw@im.ac.cn (S.L.); 2 Innovation Academy for Green Manufacture, Chinese Academy of Sciences, Beijing 100190, China; 3 University of Chinese Academy of Sciences, Beijing 100049, China; 4 Savaid Medical School, University of Chinese Academy of Sciences, Beijing 100049, China

**Keywords:** gamma-aminobutyric acid, butyrolactam, genome-scale metabolic model, metabolic rewiring, *Corynebacterium glutamicum*

## Abstract

**Simple Summary:**

The fermentative production of desired chemicals from renewable resources is one of the promising biosynthetic routes to replace the petrochemical-based process. Gamma-aminobutyric acid (GABA) can be synthesized from l-glutamic acid and used as a building block for the synthesis of butyrolactam and polyamide 4 (nylon 4). The genome-scale metabolic model can predict the growth ability and metabolic flux distribution by genetic disturbances, which provides a strategy to construct a microbial cell factory for GABA and butyrolactam biosynthesis. Here, we performed model-guided metabolic engineering of *Corynebacterium glutamicum* ATCC13032 for GABA and butyrolactam fermentation from glucose. The biosynthetic pathways of GABA and butyrolactam were constructed by overexpressing the heterologous genes using a bi-cistronic expression cassette. The genetic modifications of the metabolic network cooperatively forced the carbon flux toward GABA and butyrolactam synthesis. This study provides new insights into engineering industrial microorganisms to produce target chemicals from renewable carbon sources.

**Abstract:**

Gamma-aminobutyric acid (GABA) can be used as a bioactive component in the pharmaceutical industry and a precursor for the synthesis of butyrolactam, which functions as a monomer for the synthesis of polyamide 4 (nylon 4) with improved thermal stability and high biodegradability. The bio-based fermentation production of chemicals using microbes as a cell factory provides an alternative to replace petrochemical-based processes. Here, we performed model-guided metabolic engineering of *Corynebacterium glutamicum* for GABA and butyrolactam fermentation. A GABA biosynthetic pathway was constructed using a bi-cistronic expression cassette containing mutant glutamate decarboxylase. An in silico simulation showed that the increase in the flux from acetyl-CoA to α-ketoglutarate and the decrease in the flux from α-ketoglutarate to succinate drove more flux toward GABA biosynthesis. The TCA cycle was reconstructed by increasing the expression of *acn* and *icd* genes and deleting the *sucCD* gene. Blocking GABA catabolism and rewiring the transport system of GABA further improved GABA production. An acetyl-CoA-dependent pathway for in vivo butyrolactam biosynthesis was constructed by overexpressing *act*-encoding ß-alanine CoA transferase. In fed-batch fermentation, the engineered strains produced 23.07 g/L of GABA with a yield of 0.52 mol/mol from glucose and 4.58 g/L of butyrolactam. The metabolic engineering strategies can be used for genetic modification of industrial strains to produce target chemicals from α-ketoglutarate as a precursor, and the engineered strains will be useful to synthesize the bio-based monomer of polyamide 4 from renewable resources.

## 1. Introduction

Environmental and economic concerns drive a production mode change from the traditional petrochemical-based process to the renewable and sustainable biosynthesis route [1,2]. Amino acids as feasible sources can be produced by microbial fermentation from glucose and have been used as precursors to produce building blocks for bio-based chemicals, materials, and pharmaceuticals [3,4,5]. *Corynebacterium glutamicum* is an important industrial microbial species traditionally used for amino acid production [6], and becomes a platform organism for producing bio-based chemicals due to its robustness for fermentation and high tolerance to osmotic stress [7]. Advances in genome editing techniques and developments in metabolic engineering and synthetic biology technologies have enabled the construction of nonnative metabolic pathways to produce bio-based chemicals of industrial interests from low-cost renewable biomass in *C. glutamicum* [8,9,10,11,12,13,14,15,16], which is of great importance to reduce carbon dioxide emission and achieve carbon neutralization.

Gamma-aminobutyric acid (GABA), a nonproteinogenic amino acid, is used as a bioactive component of drugs in the pharmaceuticals and a major precursor for the synthesis of butyrolactam and polyamide 4 (nylon 4) [17,18,19,20]. GABA can be synthesized from l-glutamic acid in a one-step decarboxylation reaction catalyzed by glutamate decarboxylase (GAD, EC 4.1.1.15). The biological route of GABA synthesis has been constructed based on the GAD-catalyzed conversion of l-glutamate into GABA in a one-step or two-step process in different microorganisms [21,22,23,24,25,26]. The wild-type GAD is active at low pH values; thus, directed evolution and mutagenesis have been applied to broaden the range of GAD activity at neutral pH [27,28,29]. The heterologous expression of GAD mutants achieved the GABA biosynthesis from glucose in *C. glutamicum* [27]. The optimization of plasmid-based GAD expression by a strong promoter and an optimal ribosomal binding site as well as co-expressing *gadB1* and *gadB2* genes efficiently improved GABA production [27,30,31,32]. Chromosomal integration of multiple copies of *gdhA* and *gadB2* genes enhanced GABA production [33]. Another biological route for GABA biosynthesis from putrescine was constructed following a two-step enzymatic reaction catalyzed by *patA*-encoding putrescine aminotransferase (EC 2.6.1.82) and *patD*-encoding γ-aminobutyraldehyde dehydrogenase (EC 1.2.1.19) [34]. Genetic modifications of the metabolic network increased the carbon flux toward the GABA biosynthetic pathway. The reduction in α-ketoglutarate dehydrogenase (EC 1.2.4.2) activity by deleting *pknG* or *odhA* genes increased the supply of l-glutamate for GABA biosynthesis, and the inhibition of α-ketoglutarate dehydrogenase activity by increasing its inhibitory protein OdhI also improved putrescine-based GABA production [31,35,36]. The overexpression of PEP carboxylase (EC 4.1.1.31) and loss of malate: quinone oxidoreductase (EC 1.1.99.16) increased the OAA supply for GABA biosynthesis [37]. Blocking GABA uptake and the shunt pathway decreased GABA decomposition [38]. The secretory expression of GAD achieved efficient extracellular decarboxylation of l-glutamate [39]. In addition to glucose as a fermentation substrate, cheap carbon sources have been utilized to synthesize GABA. The overexpression of the *gadB* mutant and *xylAB* genes in *C. glutamicum* improved GABA production from the co-utilization of glucose and xylose [40]. A tunable growth-dependent bifunctional genetic switch was used to optimize the glycerol utilization pathway, which achieved the fine-tuning metabolic flux to maximize GABA production from glycerol [41]. However, maximizing GABA production from glucose is still a challenge, which needs additional engineering efforts to regulate intracellular metabolism to improve GABA production.

Butyrolactam can be used as a monomer for the production of polyamide 4, which shows improved thermal characteristics and higher hydrophobicity and biodegradability than other nylon materials [20,42]. It is also a precursor to synthesize *N*-vinylpyrrolidone, a useful solvent for injections, pharmaceuticals, and membrane filters [43]. Lactams can be easily formed by amino acids cyclization via intramolecular condensation. However, the amide bond formation by enzymatic catalysis is not thermodynamically favored in microbes, due to the endothermic high-energy requirement [44]. Consequently, the native biosynthetic pathways of lactams are unknown owing to the deficiency of enzymes capable of catalyzing the ring-cyclization step. A two-step butyrolactam biosynthetic route from l-glutamate has been reported by activation of the carboxylic group in the GABA precursor and followed by spontaneous cyclization of activated GABA [43,44,45]. The expression of GAD and acyl-CoA ligase (EC 6.2.1.3) in engineered *Escherichia coli* led to 1.1 g/L of butyrolactam from glutamate [43]. To de novo-synthesize butyrolactam from glucose, a glutamate-GABA-butyrolactam scheme was designed by co-overexpressing GAD and ß-alanine CoA transferase (EC 2.6.1.2) in the engineered *E. coli*, resulting in 435 ± 43 mg/L of butyrolactam from glucose in shake flasks [44]. A caprolactam-detecting screening system was developed to find an enzyme that cyclizes ω-amino fatty acids from marine metagenomes [46], which provides a way to rapidly screen new functional enzymes for lactam bio-production.

In this study, we performed model-guided metabolic engineering of *C. glutamicum* ATCC13032 for GABA and butyrolactam fermentation from glucose. A GABA biosynthetic pathway was constructed using a bi-cistronic expression cassette containing mutant GAD in *C. glutamicum*. Guided by a model-based simulation, the TCA cycle of *C. glutamicum* was reconstructed by increasing α-ketoglutarate synthesis and weakening α-ketoglutarate catabolism to drive more carbon flux toward the GABA biosynthetic pathway. Blocking GABA decomposition and rewiring the transport system of GABA further increased GABA production. An acetyl-CoA-dependent pathway was constructed by overexpressing *act*-encoding ß-alanine CoA transferase to synthesize butyrolactam from glucose in engineered *C. glutamicum*. This study provides new insights into engineering industrial microorganisms for the bio-based production of chemicals from renewable carbon sources.

## 2. Materials and Methods

### 2.1. Strains and Plasmids

The wild-type *C. glutamicum* ATCC13032 (American Type and Culture Collection, Manassas, VA, USA) was used as the initial strain for genetic engineering. The *E. coli* EC135 and BL21 (DE3) strains were used as the cloning and expression host, respectively [47]. The pXMJ19 was applied to overexpress the target gene with the induction of isopropylthio-β-d-galactopyranoside (IPTG) [48]. The pK18*mobsacB* was used for gene deletion, promoter replacement, and gene integration by homologous recombination events [49]. All the strains and vectors used in this study are listed in Supplementary Table S1.

### 2.2. Media and Cultivations

*E. coli* strains were cultured in Luria–Bertani medium at 37 °C for plasmid preparation. *C. glutamicum* strains were cultured in brain heart infusion medium (with 91 g/L of sorbitol) at 30 °C for competent cell preparation. If necessary, antibiotics were added at the following concentrations: 50 μg/mL of kanamycin or 34 μg/mL of chloramphenicol in *E. coli* for plasmid maintenance and 25 μg/mL of kanamycin and 10 μg/mL of chloramphenicol in *C. glutamicum* for recombinant screening.

For GABA fermentation in a shake flask, engineered *C. glutamicum* strains were precultured in the CGIII seed medium (glucose 10 g/L, yeast extract 5 g/L, peptone 10 g/L, and NaCl 10 g/L) at 30 °C for 12–14 h until the OD_600_ reached 12 [50]. Then, one milliliter of seed culture was inoculated in a 500 mL baffled shake flask with 30 mL of CGX medium consisting of glucose 40 g/L, (NH_4_)_2_SO_4_ 20 g/L, KH_2_PO_4_ 0.5 g/L, K_2_HPO_4_·3H_2_O 0.5 g/L, MgSO_4_·7H_2_O 0.25 g/L, FeSO_4_·7H_2_O 0.01 g/L, MnSO_4_·H_2_O 0.01 g/L, ZnSO_4_·7H_2_O 1 mg/L, CuSO_4_ 0.2 mg/L, NiCl_2_·6H_2_O 0.02 mg/L, and biotin 0.05 mg/L [50]. The cells were cultivated in triplicate at 30 °C and shaken at 220 rpm for 72 h in shake flasks. The samples of fermentation were harvested at 12 h intervals.

Fed-batch fermentation was carried out in a 5 L bioreactor (Shanghai bailun Bio-Technology Co, Songjiang District, Shanghai, China) with a 2 L working volume. The seed culture was carried out in 1 L shake flasks containing 200 mL of CGIII medium at 30 °C for 16–20 h to reach an optimal density of approximately 15. Then, the seed cultures were inoculated into the 5 L fermenter containing 1.8 L of CGX medium. The temperature was maintained at 30 °C using cooling water circulation. Ammonia hydroxide was used to control the pH to 7.0–7.2 during the fermentation process. The dissolved oxygen (DO) was controlled at 30% of air saturation by coupling the aeration rate and agitation speed. The glucose reservoir was fed into the fermenter at a rate of 0.2–0.5 mL/min to maintain the glucose concentration above 10 g/L in the fermentation process.

### 2.3. Genetic Manipulation

All DNA manipulations were performed using standard procedures as described previously. Primers used for PCR amplification and plasmid construction are shown in Supplementary Table S2. A codon-optimized version of *gadB* from *Lactobacillus plantarum* and ß-alanine CoA transferase from *Clostridium propionicum* were synthesized at Sangon Biotech (Shanghai, China). For overexpression of glutamate decarboxylase, *gadB* from *L. plantarum,* and *gad* and *gadM* genes from *E. coli* were amplified using the corresponding primers and were purified by the Universal DNA Purification Kit (Tiangen, Beijing, China). The PCR products together with the *Sal*I/*Bam*HI-treated pXMJ19 vector were mixed in a Gibson assembly buffer at 50 °C for 1 h and then transformed to the competent cells of EC135. To construct a vector harboring a bi-cistronic expression cassette, three DNA fragments including a *P_tuf_* promoter, the first cristron (*gsi*, *tsf*, *guaB*), and *gadM* as well as a *Eco*RV/*Bam*HI-treated pXMJ19 vector were mixed in a Gibson assembly buffer at 50 °C for 1 h and then transformed to the competent cells of EC135. To induce gene expression under the control of the *P_tac_* promoter, 1 mM isopropylthio-β-d-galactopyranoside (IPTG) was added to the culture media at 8 h after inoculation. A nonreplicating vector with a *sacB-*selectable marker was used to perform promoter replacement, gene deletion, and gene insertion in *C. glutamicum*. The pK18*mobsacB* derivatives were constructed using a Gibson assembly in EC135 (Supplementary Table S1) and then transformed into *C. glutamicum* by electroporation to screen the recombinants via two recombination events as described previously [11].

### 2.4. Constraint-Based Metabolic Flux Analysis

The genome-scale metabolic model *i*CW773 was used for in silico flux metabolic analysis of *C. glutamicum* [9]. A one-reaction synthetic route for GABA by the heterologous *gad*-encoding glutamate decarboxylase was added to *i*CW773 to synthesize GABA from glutamate. The following reaction was added to *i*CW773: ‘Glu ↔ 4ABA + CO_2_’. In silico analysis of the modified target for over-producing GABA was performed with MatLab 2014a (The MathWorks, Natick, MA, USA) and the COBRA Toolbox 2.05 (MathWorks Inc. PortolaValley, CA, USA) with the *glpk* solver [9]. Constraint-based flux balance analysis was carried out by the linear programming-based optimization of growth or GABA biosynthesis flux as a cellular objective function. The glucose uptake rate was set to 4.67 mmol/gCDW/h. Small molecular metabolites such as CO_2_, H_2_O, SO_3_, NH_3_, PO_4_, and O_2_ were allowed to be freely transported across the cell membrane. Steady-state flux solutions were solved by varying aconitase/α-ketoglutarate dehydrogenase/succinyl-CoA synthetase flux, GABA productivity, and biomass.

### 2.5. Analytical Methods

Cell concentration was determined by measuring the absorbance at 600 nm using a spectrophotometer (V-1100D; Shanghai, China). The cell dry weight (CDW) per liter was calculated following an experimentally determined formula: CDW (g/L) = 0.27 × OD_600_ [51]. Glucose concentration was measured using an SBA-40D biosensor analyzer (Institute of Biology of Shandong Province Academy of Sciences, Shandong, China).

Amino acids and GABA concentrations in the fermentation broth were determined using high-performance liquid chromatography 1200 (Agilent Technologies, Inc., Santa Clara, CA, USA) with a ZORBAX Eclipse AAA column (5 μm, 3.0 × 150 mm Agilent, Santa Clara, CA, USA) at 40 °C after online derivatization with *o*-phthalaldehyde (OPA) following the manufacturer’s protocol. For amino acid derivatization, 1 µL of sample was online-mixed with 5 µL of 0.40 M borate buffer. Following the addition of 1 µL of the OPA agent, the mixture was injected into HPLC. Mobile phase A consisted of 10 mM Na_2_HPO_4_ and Na_2_B_4_O_7_ (pH 8.2), whereas mobile phase B consisted of acetonitrile, methanol, and H_2_O (*v:v:v*, 45:45:10). The elution was performed using a gradient of mobile phase A and B at 1 mL/min. Elution gradients were: 0–0.34 min, 2% B; 0.34–13.4 min, a linear gradient of B from 2% to 57%; 13.5–15.7 min, 100% B; 15.8–18 min, 2% B. The eluate was monitored at 338 nm by a UV detector.

Acetyl-CoA and free CoA were measured using a HPLC 1200 (Agilent Technologies, Inc, Santa Clara, CA, USA) with a modified method according to the previous report [52]. An Eclipse XDB-C18 column (5 μm, 4.6 × 250 mm Agilent, Santa Clara, CA, USA) was used and operated at 25 °C. The mobile phase comprised solvent C (0.2 M sodium phosphate buffer) and solvent D (acetonitrile). The elution was performed in the mobile phase of C and D (90%/10%, *v/v*) at a constant flow rate of 1 mL/min. The eluate was monitored at 254 nm by a UV detector.

Butyrolactam was performed on a SHIMADZU LC-20AP preparative HPLC–MS system (Shimadzu (China) Co., Ltd., Xuhui District, Shanghai, China) with an Eclipse XDB-C18 column (5 μm, 4.6 × 250 mm Agilent, USA) at 25 °C. The mobile phase comprised solvent E (1% formic acid) and solvent F (methanol containing 1% formic acid). The program for HPLC was performed as per a previous report [44]. The eluent was directed to MS using electrospray ionization (ESI) positive ion mode with a 12.0 L/min drying gas flow at 350 °C and 2.5 kV capillary voltage. The scan mass range was *m/z* 50–200 kDa.

### 2.6. SDS-PAGE Analysis

The crude proteins from *C. glutamicum* cells were extracted using a lysis buffer (50 mM Tris-HCl (pH 7.5), 1 mM EDTA, 5% glycerol, 1 mM DTT) by ultrasonication. The supernatants were collected by centrifugation, reconstituted in loading buffer (1:5 ratio), and heated at 95 °C for 5 min, followed by SDS-polyacrylamide gel electrophoresis. The gel was stained with Coomassie Blue R-250 (Ge Healthcare, Chicago, IL, USA) and scanned using an EPSON Expression 11000XL at a resolution of 300 dpi.

### 2.7. RNA Preparation and Quantitative Real-Time RT-PCR

*C. glutamicum* strains were cultivated in the CGX medium as described above. The cells were harvested by centrifugation until the OD_600_ reached 20. The total RNA was isolated using the RNAprep Pure Cell/Bacteria Kit (Tiangen, China). Reverse transcription of approximately 300 ng of RNA was performed using the FastQuant RT Kit (Tiangen, China) with the specific primers listed in Supplementary Table S2. The GoTaq qPCR master mix (Promega, Madison, WI, USA) with a 20 μL volume was used to perform Quantitative PCR using the LightCycler^®^ 96 Real-Time PCR System (Roche, Basel, Switzerland). The *rpoB* gene was used as the reference gene to normalize the mRNA levels of *gltA*, *acn*, *icd*, *kgd*, *sucCD*, *aceA*, and *aceB*. Negative controls in each PCR were run to exclude DNA and other contaminants. The qPCR products were verified via a melting curve analysis. Data were analyzed using the LightCycler^®^ 96 software (Roche, Basel, Switzerland) according to the 2^−∆∆CT^ method [53].

## 3. Results

### 3.1. In Silico Simulation of Flux Distribution for GABA Overproduction

**Figure 1:**
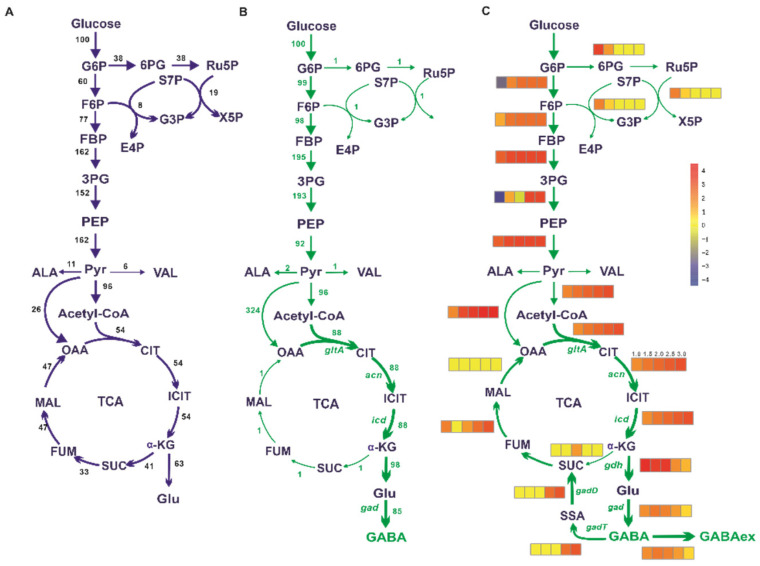
In silico simulation of the metabolic flux distribution in the WT strain and GABA overproducer by flux balance analysis. (**A**) The metabolic flux distribution in the WT strain. (**B**) The metabolic flux distribution in the GABA overproducer. The values are the respective flux to the glucose uptake rate. (**C**) The impacts of increased ACN reaction flux on intracellular metabolic flux by FBA using GABA exporter as the objective function with 20% biomass of theoretical maximum constraint. The ACN reaction flux was set to 1.0, 1.5, 2.0, 2.5, and 3.0 (from left to right) -fold compared to those in the WT strain, respectively. The corresponding changes in fluxes are shown with a heatmap of individual reaction using the normalized value. The different color represents the degree of flux changes. Yellow represents no change, red represents the increase in the flux, and blue indicates the flux toward the inverse direction as represented by the arrow.

A one-reaction synthetic route by the heterologous *gad*-encoding glutamate decarboxylase derived from *E*. *coli* was added to *i*CW773 for GABA biosynthesis. Metabolic disturbances that led to GABA overproduction were determined using flux balance analysis (FBA). The intracellular metabolic fluxes in the WT strain were calculated using the biomass as the objective function. As for the GABA producer, the lowest biomass was restricted to 20% of the theoretical maximum and the metabolic fluxes were calculated using GABA export as the objective function. The comparison of metabolic flux distribution between the WT strain and GABA producer showed the most important difference in the flux redistribution at the TCA cycle (Figure 1A,B). The flux from acetyl-CoA entering into the TCA cycle increased 63% in the GABA producer compared to the WT strain. The flux at the α-ketoglutarate node partially partitioned to succinyl-CoA for NADH generation and partially flowed to l-glutamate for biomass formation in the WT strain. In contrast, the flux from α-ketoglutarate to succinate in the GABA producer dropped to a relatively low level and a high flux of 98% was redirected toward the GABA synthetic pathway (Figure 1B and Supplementary Table S3). Considering that the *acn*-catalyzed reaction is a major control switch in the TCA cycle of *C*. *glutamicum* grown on glucose [54,55], the effects of the ACN reaction flux change on the carbon flux distribution and GABA synthesis were investigated using FBA under 20% biomass of the theoretical maximum constraint. The fluxes toward the *gltA*-catalyzed reaction and *icd*-catalyzed reaction were enhanced with the increase in ACN reaction flux. When the ACN reaction flux improved by twofold, the GABA productivity reached a relative maximum (Figure 1C). Meanwhile, the flux toward the glycolytic pathway (EMP) was increased and accompanied by a decreased flux toward the pentose phosphate pathway (PPP), which was consistent with the redistribution of carbon flux in the GABA producer (Figure 1B). When the ACN reaction flux increased beyond twofold, the flux from α-ketoglutarate to succinate showed little changes. However, the fluxes toward the GABA shunt pathway significantly increased (Figure 1C), which supplied GABA-derived succinate for TCA cycle operation but also resulted in the decrease in GABA export. Therefore, reprogramming the TCA cycle by increasing the fluxes from acetyl-CoA to α-ketoglutarate, attenuating the fluxes from α-ketoglutarate to succinate, and blocking GABA decomposition might be beneficial to GABA synthesis.

### 3.2. Constructing a GABA Biosynthetic Pathway by a Bi-Cistronic Expression Cassette

**Figure 2:**
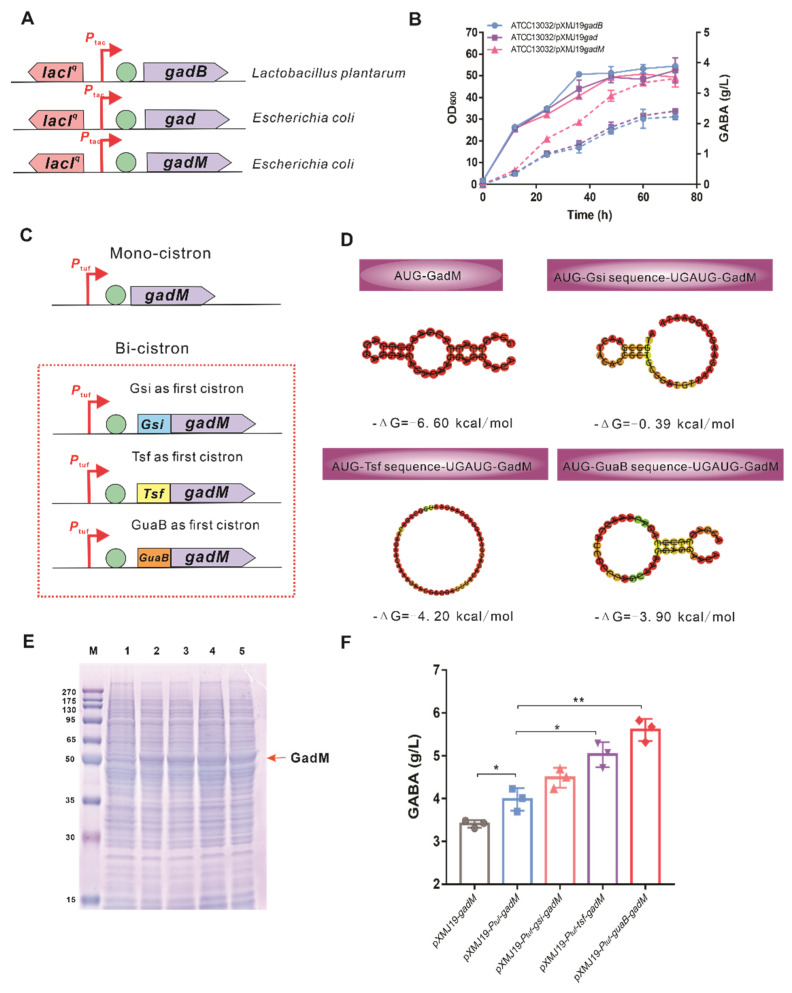
Construction and optimization of GAD-based GABA synthetic pathway. (**A**) Construction of an inducible-controlled expression cassette responsible for GABA synthesis. Three GADs encoded by *gad* and *gadM* genes from *E. coli* as well as the *gadB* gene from *Lactobacillus plantarum* were overexpressed under the *P_tac_* promoter control, respectively. (**B**) Fermentation profiles of *C*. *glutamicum* strains overexpressing different gad genes in shake flask cultivations. The solid lines represent the growth, and the dotted lines represent the GABA concentration. (**C**) The mono-cistronic and bi-cistronic expression cassettes of *GadM* under the control of a constitutive promoter *P_tuf_*. (**D**) RNAFold depiction of the mRNA secondary structures with the corresponding minimum free energy (ΔG) for mono-cistronic and bi-cistionic (*Gsi*, *Tsf*, *GuaB*-fused *GadM*) sequences. (**E**) Expression of GadM detected by SDS-PAGE in *C. glutamicum* ATCC13032. Lane 1, crude protein extract from *C. glutamicum*/pXMJ19; Lane 2, crude protein extract from *C. glutamicum*/pXMJ19-*P_tuf_*-*gadM*; Lane 3, crude protein extract from *C. glutamicum*/pXMJ19-*P_tuf_*-*gsi*-*gadM*; Lane 4, crude protein extract from *C. glutamicum*/pXMJ19-*P_tuf_*-*tsf*-*gadM*; Lane 5, crude protein extract from *C*. *glutamicum*/pXMJ19-*P_tuf_*-*guaB*-*gadM*. (**F**) GABA productions of *C*. *glutamicum* harboring different expression cassettes of *GadM* in shake flask cultivations. Data are presented as mean values with the standard deviation from three biological replicates. Significant differences were determined using Student’s *t* test (* *p* < 0.05, ** *p* < 0.01).

To construct the GABA synthetic pathway, *gad* and its mutant *gadM* from *E*. *coli* as well as *gadB* from *L. plantarum* were overexpressed under the inducible *P_tac_* promoter in *C*. *glutamicum* ATCC13032 (Figure 2A). The effect of those enzymes on GABA production was determined in shaking flasks. As shown in Figure 2B, the expression of *gadM* resulted in the accumulation of 3.48 g/L of GABA, approximately 1.56- and 1.44-fold higher than those by *gadB* and *gad*-encoding GAD, respectively, which is consistent with the previous report that *GadM* from *E. coli* has the potential to produce a high titer of GABA in *C*. *glutamicum* [40].

To further increase the production of GABA, the expression of heterogenous GadM was optimized at the transcriptional and translational levels, respectively. To increase the mRNA level of *gadM*, a mono-cistronic expression cassette was designed and constructed using a constitutive strong *P_tuf_* promoter (Figure 2C). The resultant ATCC13032/pXMJ19-*P_tuf_* -*gadM* strain produced 4.05 ± 0.09 g/L of GABA, which increased by 16% compared to the ATCC13032/pXMJ19-*gadM* strain. The ribosome’s interactions with the 5’-terminus around the RBS and the start codon play an important role in controlling the translation initiation rate of protein; thus, the mRNA secondary structure in codons corresponding to the first 16 amino acids at the N-terminus affects the protein abundance [56,57,58]. The secondary structure of the GadM mRNA sequence at the N-terminus was analyzed by the RNAfold tool [59]. As shown in Figure 2, the rigid stem-loop structure in the 5’-terminus might block ribosome binding, resulting in translation attenuation. To increase the translation initiation rate of GadM, the first 15 amino acid sequences of *gsi*, *tsf*, and *guaB* genes encoding proteins with a high abundance were used as the first cistron in a bi-cistronic expression cassette to reduce the intramolecular secondary structure (Figure 2C). The RNA folding predictions showed that the 5’-terminus secondary structure shared an open conformation and the minimum free energies (ΔG) of three bi-cistronic sequences were higher than that of the mono-cistronic sequence (Figure 2D). The more destabilized ΔG indicated that the start codon of GadM could be accessible to RNA polymerase to initiate the translation. The effects of bi-cistronic expression cassettes on the abundance of GadM were determined by SDS-PAGE (Figure 2E). The expression level of GadM in three bi-cistronic expression cassettes was a little higher than that in a mono-cistronic expression cassette. As expected, GABA production increased in the ATCC13032 strains harboring three bi-cistronic expression cassettes (Figure 2F), in which ATCC13032/pXMJ19-*P_tuf_*-*guaB*-*gadM* (GABA-1 strain) produced 5.60 ± 0.26 g/L of GABA with a 38% increase over ATCC13032/pXMJ19-*P_tuf_*-*gadM*.

### 3.3. Reprogramming TCA Cycle to Improve GABA Production

**Figure 3:**
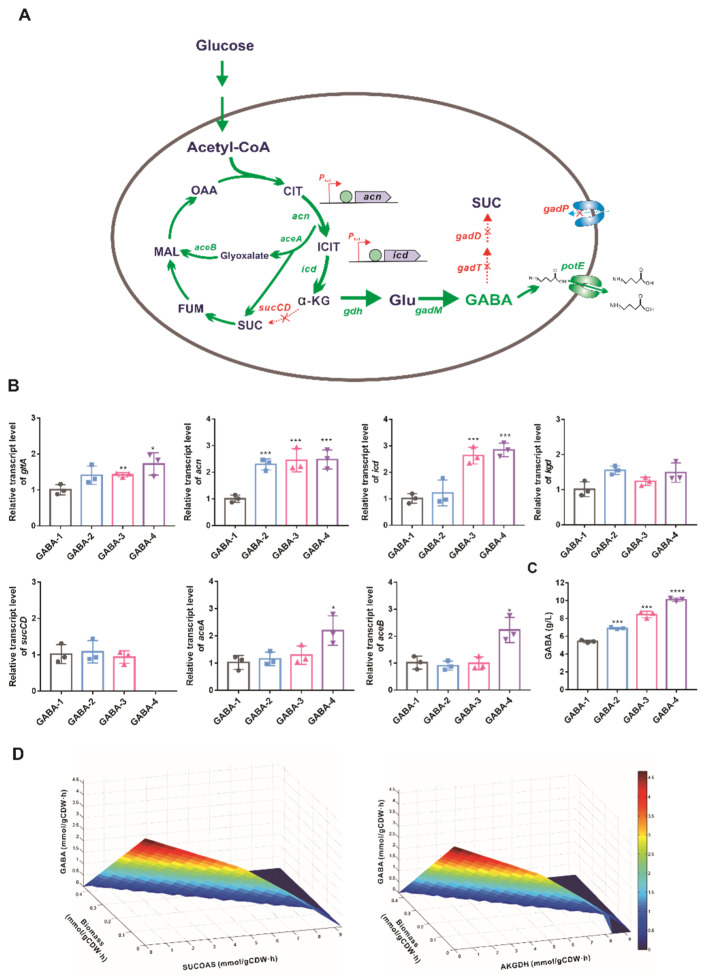
Reprogramming TCA cycle for GABA production. (**A**) The strategy for reprogramming the metabolic network for GABA biosynthesis. The red X represents the deletion of the corresponding gene. The green arrows indicate increased flux by gene overexpression. The enzymes encoded by the genes shown here are *acn*, aconitase; *icd*, isocitrate dehydrogenase; *sucCD*, succinyl-CoA synthetase; *gdh*, glutamate dehydrogenase; *gadM*, glutamate decarboxylase; *gadT*, 4-aminobutyrate-2-oxoglutarate transaminase; *gadD*, succinate-semialdehyde dehydrogenase; *gadP*, 4-aminobutyrate transporter; *potE*, bi-directional cadaverine transporter. Abbreviations: CIT, citrate; ICIT, isocitrate; α-KG, α-ketoglutarate; SUC, succinate; FUM, fumaric acid; MAL, malate; OAA, oxaloacetate; Glu, glutamate; GABA, gamma-aminobutyric acid. (**B**) Relative transcript levels of related genes of the GABA strains in the shake flask cultivations. (**C**) GABA productions in the shake flask cultivations of the engineered strains. (**D**) Simulation of the impact of AKGDH and SUCOAS fluxes on GABA productivity. Steady-state flux solutions were obtained using the GABA productivity as an objective function in combination with varying the AKGDH/SUCOAS flux and the growth rates. Data are presented by mean values with the standard deviation from three biological replicates. Significant differences were determined using Student’s *t* test (* *p* < 0.05, ** *p* < 0.01, *** *p* < 0.001, **** *p* < 0.0001).

Guided by model-predicted metabolic flux distributions, the TCA cycle of *C*. *glutamicum* ATCC13032 was reconstructed by increasing the synthesis of α-ketoglutarate and weakening the catabolism of α-ketoglutarate (Figure 3A). In the in silico simulation, the relative ACN reaction flux improved by twofold, resulting in a significant increase in GABA productivity. In our previous studies, three promoters of varying strength (*P_glyA_*, *P_pck_*, and *P_tuf_*) had been used to regulate the transcription of the *acn* gene, and the mRNA levels of the *acn* gene mediated by *P_tuf_* increased by 2.83-fold [9]. Therefore, the native promoter of the *acn* gene was replaced by the *P_tuf_* promoter. The resultant GABA-2 strain produced 6.90 ± 0.08 g/L of GABA with a 27% increase over the GABA-1 strain. With upregulation of the *acn* gene, the mRNA levels of *gltA* in GABA-2 increased by 40%, which might attribute to a similar regulatory mechanism of *gltA* and *acn* transcription [55]. However, no significant changes were observed in the mRNA level of *icd* (Figure 3B). To drive more flux from isocitrate to α-ketoglutarate, the *P_tuf_* promoter was used to control the expression of the *icd* gene. The mRNA level of *icd* was increased by 2.5-fold, while there were no changes in mRNA levels of *kgd* and *sucCD* genes in the GABA-3 strain. As expected, GABA-3 produced 8.44 ± 0.30 g/L of GABA with a 56% increase over the GABA-1 strain (Figure 3C).

The catabolism of α-ketoglutarate to succinate is sequentially catalyzed by α-ketoglutarate dehydrogenase complex (AKGDH, EC 1.2.4.2) and *sucCD*-encoding succinyl-CoA synthetase (SUCOAS, EC 6.2.1.5) in *C*. *glutamicum* [60]. The relationship between the flux toward AKGDH (or SUCOAS), GABA productivity, and growth rate was investigated by double-robustness analysis. As taking GABA productivity as an optimal criterion, the biomass and the flux through AKGDH and SUCOAS decreased to zero with maximizing GABA productivity (Figure 3D). Considering that succinyl-CoA generated from AKGDH is essential for biomass formation and peptidoglycan synthesis in *C*. *glutamicum* [61], the inactivation of succinyl-CoA synthetase by deleting *sucCD* genes was performed to construct the GABA-4 strain. In the shake flasks, the specific growth rate of the GABA-4 strain was comparable to those of GABA-2 and GABA-3 strains (Supplementary Figure S1), indicating that shutting off the flux from succinyl-CoA to succinate might have little effect on the operation of the TCA cycle. In the GABA-4 strain, the mRNA levels of *aceA* and *aceB* increased by twofold (Figure 3C), suggesting that the glyoxylate cycle was activated to maintain the growth of the GABA-4 strain. Finally, GABA production improved to 10.1 ± 0.18 g/L.

### 3.4. Blocking Decomposition and Rewiring Transport of GABA

**Figure 4:**
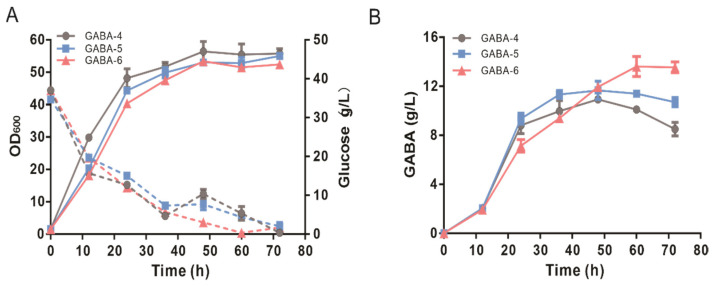
The effects of blocking the GABA shunt pathway and rewiring the GABA transport system on GABA production. (**A**) Time profiles of cell growth and glucose concentration during the shake flasks. The solid lines represent the growth, and the dotted lines represent the glucose concentration. (**B**) Time profile of GABA production during the shake flasks. Data shown are mean values with the standard deviation from three biological replicates.

In the shake flask cultivation of the GABA-4 strain, the decrease in GABA concentration after fermentation for 60 h indicated that GABA might be catabolized to succinate via the GABA shunt pathway or re-uptake into cells (Figure 4A) [38]. The GABA shunt pathway consists of the conversion of GABA to succinic semialdehyde by *gadT* encoding 4-aminobutyrate aminotransferase (EC 2.6.1.19) followed by the conversion of succinic semialdehyde to succinate by *gadD*-encoding succinate-semialdehyde dehydrogenase (EC 1.2.1.24) (Figure 3A). To decrease the decomposition of GABA, the GABA shunt pathway was blocked by deleting *gadT* and *gadD* genes. In the resultant GABA-5 strain, GABA increased continuously from 12 h to 36 h and then slightly decreased from 60 to 72 h (Figure 4B). The GABA decrement at the final stage of GABA-5 fermentation was 38% lower than that of the GABA-4 strain.

Blocking the uptake of GABA and promoting the excretion of GABA will decrease the intracellular GABA pool and consequently lead to the enhancement of the GABA production. It has been reported that the deletion of the *gadC* gene encoding glutamic acid:4ABA antiporter has little effect on GABA accumulation [44]. The *gadP* gene encoding GABA:H^+^ symporter had been identified as a major transport for the uptake of GABA, and the overexpression of GadP led to the re-uptake of GABA [44,62]. However, the specific exporter responsible for GABA excretion has not been identified. PotE is a bi-directional putrescine and cadaverine export in *E. coli* [63,64]. GABA might be exported by PotE due to the similar structures between GABA and putrescine. Therefore, *potE* was integrated into the genome of the GABA-5 strain for the replacement of the *gadP* gene to rewire the transport system of GABA. The resultant GABA-6 strain produced 13.62 ± 1.42 g/L of GABA, which was 17% higher than that of the GABA-5 strain (Figure 4B). Compared with the GABA-4 strain, blocking the GABA shunt pathway in the GABA-5 stain and further rewiring the transport system in the GABA-6 strain led to an increase in average productivity of GABA (0.11 ± 0.01 and 0.13 ± 0.01 vs. 0.09 ± 0.00 mmol/gCDW/h, respectively).

### 3.5. Construction of a Metabolic Pathway for Butyrolactam Biosynthesis

**Figure 5:**
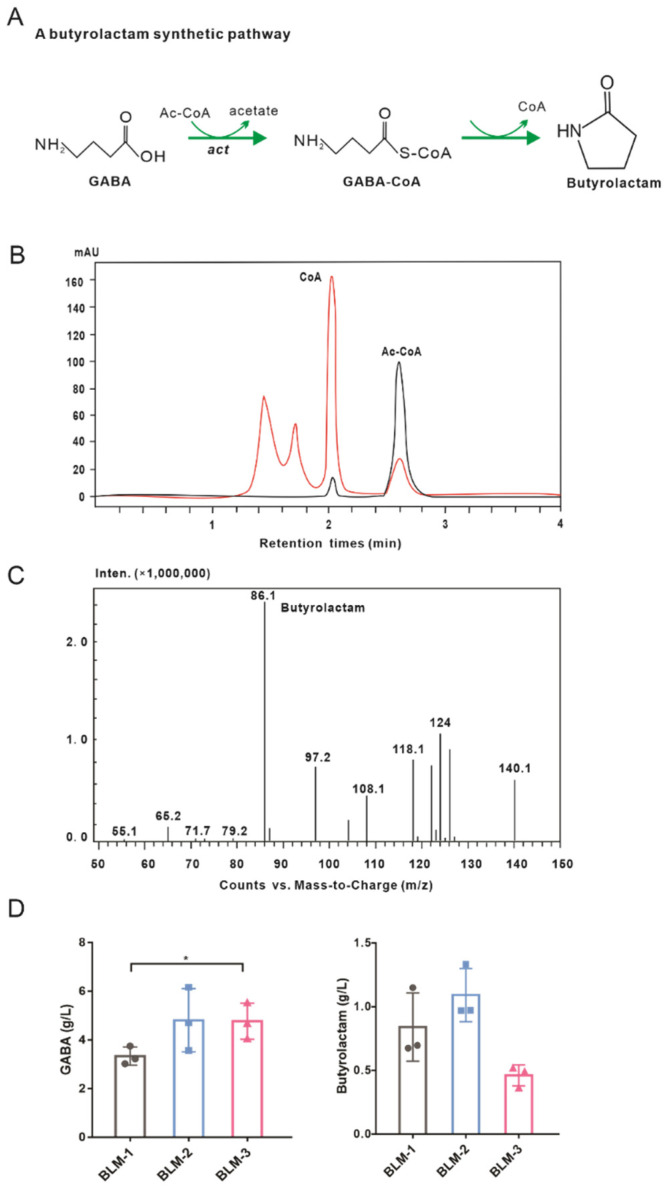
A metabolic pathway for butyrolactam biosynthesis. (**A**) An acetyl-CoA-dependent pathway for butyrolactam biosynthesis from GABA. (**B**) In vitro analysis of Act activity using HPLC. Time profiles of CoA and acetyl-CoA concentrations are shown. Black represents negative control and red represents experimental group assay. (**C**) Analysis of butyrolactam production by the recombinant BLM-1 strain in flask cultivation using HPLC–MS. (**D**) GABA and butyrolactam productions from glucose in the engineered BLM strains. Data shown are mean values from three biological replicates and the standard deviations are presented. Significant differences were determined using Student’s *t* test (* *p* < 0.05).

As previously reported, ß-alanine CoA transferase can catalyze the spontaneous cyclization of GABA to butyrolactam (Figure 5A) [44]. The *act*-encoding ß-alanine CoA transferase derived from *C. propionicum* was overexpressed in *E. coli* BL21 by IPTG induction, and the crude proteins were extracted to detect Act activity using GABA and acetyl-CoA as substrates. The free CoA level was significantly increased and the AcCoA level was decreased compared to the negative control (Figure 5B), indicating that free CoAs might be released due to spontaneous cyclization of GABA to butyrolactam. However, GABA-CoA was not detected using HPLC–MS, which might attribute to the rapid conversion to butyrolactam as observed previously [44]. The in vitro enzymatic assay verified the function of *act*-encoding ß-alanine CoA transferase.

Considering that GABA is the precursor for butyrolactam, engineered GABA strains were used as chassis cells for butyrolactam biosynthesis. Therefore, we constructed an acetyl-CoA-dependent pathway for butyrolactam biosynthesis in vivo by co-overexpressing *act* and *gadM* genes in WT-*P_tuf_*-*acn*-*P_tuf_*-*icdΔsucCD,* WT-*P_tuf_*-*acn*-*P_tuf_*-*icdΔsucCDΔgabDT,* and WT-*P_tuf_*-*acn*-*P_tuf_*-*icdΔsucCDΔgabDTΔgabP*::*potE*, resulting in BLM-1, BLM-2, and BLM-3 strains. As expected, butyrolactam could be detected in the flask cultivation using HPLC–MS (Figure 5C and Supplementary Figure S2). The BLM-1 strain produced 0.84 ± 0.26 g/L of butyrolactam. As the conversion of GABA in vivo was blocked, butyrolactam production in the BLM-2 strain increased to 1.09 ± 0.21 g/L, which was 31% higher than that in the BLM-1 strain (Figure 5D). However, a decrease in butyrolactam concentration was observed in the BLM-3 strain. We deduced that blocking GABA import and improving GABA export in the BLM-3 strain might cause the decrease in intracellular GABA pool, resulting in a decrease in precursor supply for butyrolactam biosynthesis.

### 3.6. The Performance of Engineered GABA-6 and BLM-2 Strains in Fed-Batch Fermentation

**Figure 6:**
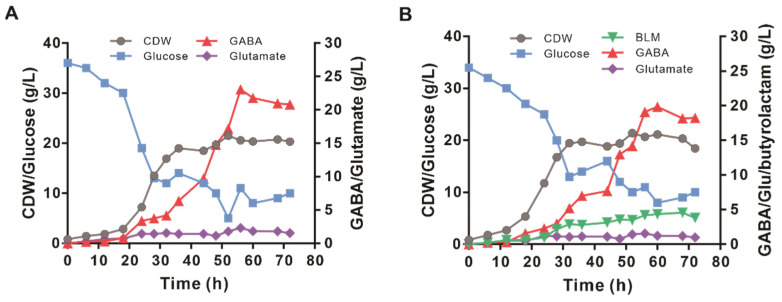
Fed-batch fermentation profiles of the engineered GABA and butyrolactam-producing strains in a 5 L bioreactor. (**A**) Time profiles of cell growth, glucose, glutamate, and GABA concentrations during the fed-batch cultivation of GABA-6 strain are shown. (**B**) Time profiles of cell growth, glucose, glutamate, GABA, and butyrolactam concentrations during the fed-batch cultivation of BLM-2 strain are shown.

The production performance of the GABA-6 and BLM-2 strains was investigated in fed-batch fermentation with minimal medium. As shown in Figure 6A, the GABA-6 strain grew continuously from 0 h to 36 h and reached a maximal cell dry weight of 20.74 gCDW/L. GABA began to accumulate at 12 h and continuously increased in the fermentation period. In the feeding phase, the maximal productivity of GABA reached 0.66 g/L/h. The specific growth rate of strain GABA-6 was 0.16 1/h, and the glucose consumption rate was 7.1 mmol/L/h. The maximal GABA titer reached 23.07 g/L at 56 h with a yield of 0.52 mol/mol of glucose and an average productivity of 0.38 g/L/h. The glutamate concentration was maintained at a relatively low level during the whole fermentation. As for the BLM-2 strain, the maximal butyrolactam production reached up to 4.58 g/L at 68 h (Figure 6B). However, the GABA production was higher than that of butyrolactam during the whole fermentation process, and 18.24 g/L of GABA was accumulated at the end of fermentation (Figure 6B).

## 4. Discussion

The overexpression of heterologous genes in chassis cells is commonly used to construct a new synthetic pathway or redirect metabolic flux into the synthetic pathway of desired metabolites. The expression levels of heterologous genes are determined by multiple factors, such as gene dosage, promoter strength, secondary structure of mRNA, RBS, and translation initiation efficiency [65,66]. For the one-step decarboxylation catalyzed by the *gad* gene, GABA production is mostly dependent on the abundance of GAD; therefore, many efforts have been adopted to increase the expression level of GAD [27,30,32]. Consistent with previous reports, the strong *P_tuf_* promoter was used to mediate the constitutive expression of *gadM*, resulting in a 16% increase in GABA production. The mRNA structure determines the accessibility of ribosome to translation initiation; thus, a high degree of mRNA structure at the N-terminus can be unavailable for ribosome binding to initiate translation [59]. Using a bi-cistronic expression cassette to optimize mRNA structure, an open conformation of GAD with a higher ΔG value strengthened the recruitment and binding of ribosomes to RBS, resulting in the increase in the translation efficiency of the GAD protein and GABA production. Our results indicate that a bi-cistronic expression cassette with a destabilized mRNA structure contributes to improve the expression level of a heterologous gene.

Metabolic reprogramming has been performed by taking advantage of biotechnologies to overcome the metabolic bottleneck in product synthesis. Guided by in silico model-based simulation, we reprogrammed the metabolic network for GABA production by increasing the α-ketoglutarate supply, blocking GABA catabolism, and rewiring GABA transport. Precursor α-ketoglutarate is crucial to GABA production; however, most α-ketoglutarate is converted to succinyl-CoA for peptidoglycan biosynthesis by AKGDH and succinate by SUCOAS for the operation of the TCA cycle. AKGDH is composed of three subunits encoded by *odhA*, *sucB,* and *lpdA* genes, and its activity is regulated by PknG and OdhI [67]. Metabolic engineering strategies focused on reducing the α-ketoglutarate dehydrogenase activity by deleting *odhA/pknG* or overexpressing *odhI* genes for GABA production [31,35]. Unlike previous reports, we first pushed more carbon flux toward α-ketoglutarate synthesis by increasing the expression of *acn* and *icd* genes, and then redirected α-ketoglutarate toward GABA synthesis by deleting the *sucCD* gene rather than inhibiting AKGDH activity. SucCD deficiency increased GABA production, demonstrating that the blocking of the conversion of succinyl-CoA to succinate led to the increase in α-ketoglutarate pool for GABA synthesis. There was no obvious effect of *sucCD* deficiency on the cell growth of engineered *C. glutamicum*, which might attribute to two aspects. Unlike the inhibition of α-ketoglutarate dehydrogenase activity, the deficiency of *sucCD* does not significantly impact the synthesis of succinyl-CoA, which is available for peptidoglycan and wall biosynthesis to support cell growth [61]. On the other hand, the increase in the transcription of *aceA* and *aceB* genes indicated that the deficiency of *sucCD* activated the glyoxylate shunt as a partial by-pass route for succinate formation to support cell growth; however, the glyoxylate shunt pathway was off in the *C. glutamicum* grown on glucose [60]. Similarly, the deletion of *sucCD* in 4-hydroxyproline-producing *C. glutamicum* drove the redirection of carbon flux partitioning at the α-ketoglutarate node and had no significant impact on the cellular growth [68]. In addition, no elevated overflow of flux in response to *sucCD* deletion in *C. glutamicum* indicated that the TCA cycle could normally operate as the inactivation of succinyl-CoA synthetase [60]. Other reported strategies for TCA cycle modification by deleting *pknG* or *odhA,* deleting *mdh,* and overexpressing *ppc* had a positive effect on GABA biosynthesis via the GAD-based route, and GABA production improved to 31.1 g/L, 29.5 g/L, and 26.3 g/L, respectively [31,35,37]. The GABA-6 strain shows a similar potential for GABA production, demonstrating the effectiveness of genetic modification of the TCA cycle in this study. The strategy used in this study has a potential value for reprogramming the TCA cycle to produce target chemicals from α-ketoglutarate as a precursor. As for the GABA synthesis via the putrescine route, increasing the precursor supply together with blocking the decomposition and rewiring the transporter resulted in a significant improvement of GABA titer (63.2 g/L) [36]. In contrast, the removal of the above limitations for GABA synthesis via the GAD-based route had no significant effects on GABA titer as expected, indicating that GAD activity is still a key limiting factor for GABA production. Even if the pH threshold of GAD was broadened to 6.0 by deleting the C-terminal [28,29], the glutamate and GABA would not be synthesized simultaneously, due to the occurrence of glutamate biosynthesis at pH 6.7–7.5 [33]. Therefore, directed evolution of GAD to improve its enzymatic activity at neutral pH or two-stage fermentation based on the pH control will contribute to improve GABA production from glucose via the GAD-based biosynthetic route.

The microbial fermentation production of lactams is relatively difficult due to the lack of natural biosynthetic pathways and catalytic enzymes. Despite an Act-mediated synthetic pathway being constructed, it showed a low efficiency for butyrolactam production [43,45]. Due to the broader activities of Act on various ω-amino acids, Act catalyzes GABA, 5-aminovaleric acid, and 6-aminocaproic acid to generate butyrolactam, valerolactam, and caprolactam, which might result in the co-production of lactam [44]. In our engineered *C. glutamicum*, only butyrolactam was detected and none of valerolactam and caprolactam were co-accumulated owing to the incapacity of the engineered strain to synthesize 5-aminovaleric acid and 6-aminocaproic acid. However, GABA production was higher than butyrolactam production in all engineered BLM strains and in fed-batch fermentation, indicating that the activity of ß-alanine CoA transferase might be a limiting factor for acetyl-CoA-dependent butyrolactam biosynthesis. Screening new lactam-synthesizing enzymes with a high catalytic efficiency can provide the biological element with a high catalytic efficiency for metabolic engineering of lactam bio-production.

## 5. Conclusions

In this study, we constructed engineered *C. glutamicum* for GABA and butyrolactam fermentation from glucose guided by in silico simulation. The biosynthetic pathways of GABA and butyrolactam were constructed by overexpressing heterologous genes using a bi-cistronic expression cassette. The genetic modifications of genes cooperatively forced the carbon flux toward GABA and butyrolactam synthesis. This work provides new insights into engineering industrial microorganisms for the bio-based production of chemicals from renewable carbon sources.

## Data Availability

All data used or analyzed during this study are included in this article and the Supplementary Materials.

## Supplementary Materials

The following supporting information can be downloaded at: https://www.mdpi.com/article/10.3390/biology11060846/s1, Figure S1. The growth profiles of engineered *C. glutamicum* strains harboring pXMJ19-*P_tuf_*-*guaB-gadM* in shake flask cultivations; Figure S2. The analysis of butyrolactam in the shake flask cultivation using HPLC-MS. Table S1. Strains and plasmids used in this study; Table S2. Primers used in this study; Table S3. Fluxes calculated by FAB in the WT strain and the GABA producer References [9,47,48,49,68] are cited in the supplementary materials.
