# Supplementary material for: Model-Guided Metabolic Rewiring for Gamma-Aminobutyric Acid and Butyrolactam Biosynthesis in Corynebacterium glutamicum ATCC13032
Source: Biology (Basel). 2022 May 31;11(6):846. doi: 10.3390/biology11060846 (PMC9219837; doi:10.3390/biology11060846)
Supplement: Supplementary file 1 [file biology-11-00846-s001.zip › biology-1715972-Supplementary.pdf]

Supplementary Materials

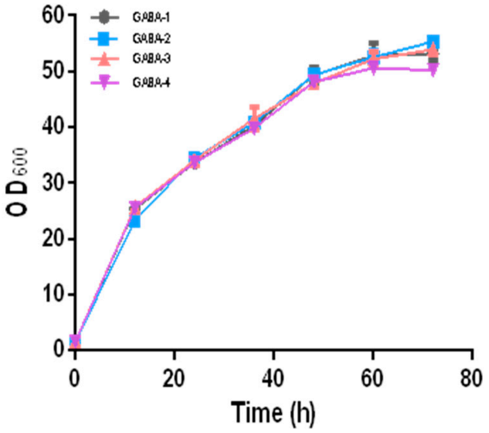

Figure S1. The growth profiles of engineered *C. glutamicum* strains harboring pXMJ19-P_{tuf}-*guaB-gadM* in shake flask cultivations.

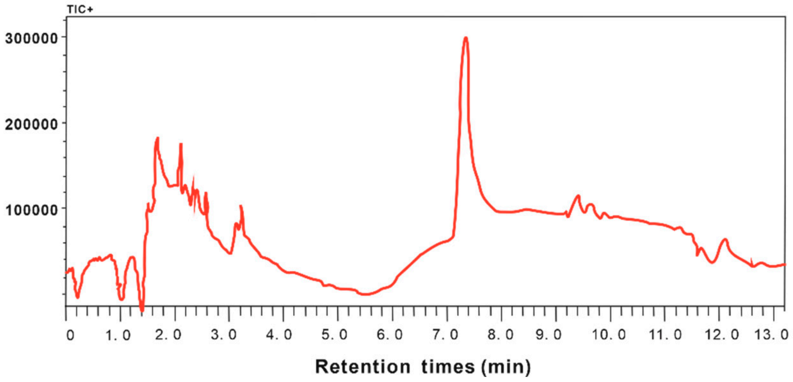

Figure S2. The analysis of butyrolactam in the shake flask cultivation using HPLC-MS. Time profiles of fermentation sample are shown.

Table S1. Strains and plasmids used in this study.

**Strains**	Characteristics	Source
E. coli		
EC135	TOP10*Δdcm::FRTrecA*$^+$*Δdam::FRT*, genotype of R-M systems:	(Zhang et. al., 2012)
BL21(DE3)	*mcrAΔ(mrr-hsdRMS-mcrBC)Δdcm::FRTΔdam::FRT*	
C. glutamicum	F$^-$ *ompT gal dcm lon hsd*S$_B$(r$_B^-$ m$_B^-$) λ(DE3)	Novagen
ATCC 13032	wild-type, biotin-auxotrophic	ATCC
GABA-1	13032 harboring pXMJ19-P_{tuf}-*guaB-gadM*	This study
GABA-2	WT-P_{tuf}-*acn* harboring pXMJ19-P_{tuf}-*guaB-gadM*	This study
GABA-3	WT-P_{tuf}-*acn*-P_{tuf}-*icd* harboring pXMJ19-P_{tuf}-*guaB-gadM*	This study
GABA-4	WT-P_{tuf}-*acn*-P_{tuf}-*icdΔsucCD* harboring pXMJ19-P_{tuf}-*guaB-gadM*	This study
GABA-5	WT-P_{tuf}-*acn*-P_{tuf}-*icdΔsucCDΔgabDT* harboring pXMJ19-P_{tuf}-*guaB-gadM*	This study
GABA-6	WT-P_{tuf}-*acn*-P_{tuf}-*icdΔsucCDΔgabDΔgabP*::*potE* harboring pXMJ19-P_{tuf}-*guaB-gadM*	This study
BLM-0	13032 harboring pXMJ19	This study
BLM-1	WT-P_{tuf}-*acn*-P_{tuf}-*icdΔsucCD* harboring pXMJ19-P_{tuf}-*guaB-gadM-act*	This study
BLM-2	WT-P_{tuf}-*acn*-P_{tuf}-*icdΔsucCDΔgabDT* harboring pXMJ19-P_{tuf}-*guaB-gadM-act*	This study
BLM-3	WT-P_{tuf}-*acn*-P_{tuf}-*icdΔsucCDΔgabDTΔgabP*::*potE* harboring pXMJ19-P_{tuf}-*guaB-gadM-act*	This study
**Plasmids**		
pK18*mobsacB*	Mobilizable vector, allows for selection of double crossover in *C. glutamicum*, *Kan*^{r}	(Schafer et al., 1994)
pK18*mobsacB*-P_{tuf}-*acn*	pK18*mobsacB* derivate carrying P_{tuf}-*acn* replacement	(Zhang et al., 2017)
pK18*mobsacB*-P_{tuf}-*icd*	pK18*mobsacB* derivate carrying P_{tuf}-*icd* replacement	This study
pK18*mobsacB-ΔsucCD*	pK18*mobsacB* derivate carrying *sucCD* deletion	(Zhang et al., 2019)
pK18*mobsacB-ΔgabDT*	pK18*mobsacB* derivate carrying *gabDT* deletion	This study
pK18*mobsacBΔgabP*::*potE*	pK18*mobsacB* derivate carrying *gabP* deletion and *potE* integration	This study
pXMJ19	Shuttle vector (Cm^r P_{tac} *lacI*^{q} *pBL1 oriV*$_{C. glutamicum}$ *pK18 oriV*$_{E. coli}$)	(Jakoby et al.,1999)
pXMJ19-*gadB*	pXMJ19 derivate carrying P_{tac}-*gadB*	This study
pXMJ19-*gad*	pXMJ19 derivate carrying P_{tac}-*gad*	This study
pXMJ19-*gadM*	pXMJ19 derivate carrying P_{tac}-*gadM*	This study
pXMJ19-P_{tuf}-*gadM*	pXMJ19 derivate carrying P_{tuf}-*gadM*	This study
pXMJ19-P_{tuf}-*tsf-gadM*	pXMJ19 derivate carrying P_{tuf}-*tsf-gadM*	This study
pXMJ19-P_{tuf}-*gsi-gadM*	pXMJ19 derivate carrying P_{tuf}-*gsi-gadM*	This study
pXMJ19-P_{tuf}-*guaB-gadM*	pXMJ19 derivate carrying P_{tuf}-*guaB-gadM*	This study
pXMJ19-P_{tuf}-*guaB-gadM-act*	pXMJ19 derivate carrying P_{tuf}-*guaB-gadM-act*	This study
pET-28a	Kan^r; expression vector with an N-terminal hexahistidine affinity tag	Novagen
pET-28a-*act*	pET-28a derivate carrying *act* gene	This study

Table S2. Primers used in this study.

Primers	Sequence (5'-3')	Note
WZ4128	GCTTGCATGCCTGCAGGTCGACATGGAAGATGTTCACATGCTGTACGGT	*gadB* amplification to construct pXMJ19-*gadB*
WZ4129	GAGCTCGGTACCCGGGGATCCTCAGTGGGTGAAGCCGTAGGTTTTGT	
WZ4132	TAAGCTTGCATGCCTGCAGGTCGACATGGATAAGAAGCAAGTAACGGATTTAAGGT	*gad* amplification to construct pXMJ19-*gad*
WZ4133	AATTCGAGCTCGGTACCCGGGGATCCTCAGGTATGTTTAAAGCTGTTCTGTTGGGCAAT	
WZ4134	TAAGCTTGCATGCCTGCAGGTCGACATGGATAAGAAGCAAGTAACGGATTTAAGGT	*gadM* amplification to construct pXMJ19-*gadM*
WZ4135	AATTCGAGCTCGGTACCCGGGGATCCTCAGTGATCGCTGAGATATTTCAGGGAAGCT	
WZ4136	AATTAAGCTTGCATGCCTGCAGGTCGACATGGATAAGAAGCAAGTAACGGAT	*gadM* amplification to construct
WZ4137	AATTAAGCTTGCATGCCTGCAGGTCGACATGGATAAGAAGCAAGTAACGGAT	pXMJ19-P_{tuf}-*gadM*
WZ4138	GTCGTATCCCACTACCGAGATTGGCCGTTACCCTGCGAATGT	P_{tuf} amplification to construct
WZ4139	GTCGACCTGCAGGCATGCAAGCTTAATTTGTATGTCCTCCTGGACTTCGT	pXMJ19-P_{tuf}-*gadM*
WZ4141	TGCGGATGTTAAGAAGGAGGAATAAATGGATAAGAAGCAAGTAACGGAT	*gadM* amplification to construct pXMJ19-*P_{tuf}-tsf-gadM*
WZ4137	AATTAAGCTTGCATGCCTGCAGGTCGACATGGATAAGAAGCAAGTAACGGAT	
WZ4142	AATTAAGCTTGCATGCCTGCAGGTCGACATGGCGAACTACACCGCTGCGG	*tsf* amplification
WZ4143	TTATTCCTCCTTCTTAACATCCGCAGCGGTGTAGTTCGCCAT	
WZ4144	AATAACAAAATAAGGAGGATTTACATATGAATGGATAAGAAGCAAGTAACGGAT	*gadM* amplification to construct pXMJ19-*P_{tuf}-gsi-gad*
WZ4137	AATTAAGCTTGCATGCCTGCAGGTCGACATGGATAAGAAGCAAGTAACGGAT	
WZ4145	AATTAAGCTTGCATGCCTGCAGGTCGACATGGCAGACAATAACAAA	*gsi* amplification
WZ4146	TTCATATGTAAATCCTCCTTATTTTGTTATTGTCTGCCAT	
WZ4147	ATCGCCCAGTAAAGGAGGAATAAATGGATAAGAAGCAAGTAACGGAT	*gadM* amplification to construct pXMJ19-*P_{tuf}-guaB-gadM*
WZ4137	AATTAAGCTTGCATGCCTGCAGGTCGACATGGATAAGAAGCAAGTAACGGAT	
WZ4148	AAGGAGGATCGCCCCGTAATGAGCCTTCAGACAAATCATC	*guaB* amplification
WZ4149	TTATTCCTCCTTTACTGGGCGATGATTTGTCTGAAGGCTCAT	
WZ4150	CCGGAATTCAAAATCTGATTCCTTTGCA	

Primers	Sequence (5'-3')	Note
WZ4151	TTCGCAGGGTAACGGCCACTTCATTATCCTAACAGTAC	Replacement of *acn* promoter with P_{tuf}
WZ4152	GTACTGTTAGGATAATGAAGTGGCCGTTACCCTGCGA	
WZ4153	AGTCACAGTGAGCTCCATTTCTATCCTCCTTTTGTATGTCCTCCTG	
WZ4154	ATACAAAAGGAGGATAGAAATGGAGCTCACTGCCT	
WZ4155	CCCAAGCTTTGGTGGTGTGGGAGTCG	
WZ4158	ATGTGCTGCAAGGCGATTAA	
WZ4159	TATGCTTCCGGCTCGTATGT	
WZ4160	TTGCATGCCTGCAGGTCGACGCGGTGTGGGAACTTCTTAA	Replacement of *icd* promoter with P_{tuf}
WZ4161	GAGCCCATCAACCAAGGAGACTCATGGCTAAGATCATCTGGAC	
WZ4162	GAGTCTCCTTGGTTGATGGGCTCTGTATGTCCTCCTGGACTTC	
WZ4163	ACTGTATTCTAGGTAGCTGAACAAAATGGCCGTTACCCTGCGAAT	
WZ4164	TTTGTTCAGCTACCTAGAATACAGTGTTCTACTAATTGCTGGCGCCTA	
WZ4165	ATTCGAGCTCGGTACCCGGGGATCCATGAAACCGCAGCACCCGCAAT	
WZ4166	TGCATCCATGGTTGCAACGTT	
WZ4167	AACCGCAGCACCCGCAATCGCGCGCATCCTCGAA	
WZ4176	ACAGCTATGACATGATTACGAATTCTGTTAAAGACGCAGAAGGCTCT	*sucCD* knockout
WZ4177	TCAGTAATAATCACGCACAGTGTGTCCTCATCAATACCAGTGAG	
WZ4178	CACTGTGCGTGATTATTACTGA	
WZ4179	CCTGCAGGTCGACTCTAGAGGATCCAGTGCCTTCTGAACCTGTCAC	
WZ4180	GTCCTGCGCACAGATGAATACTCT	
WZ4181	GCTGAGCACCACGGATCCAAT	
WZ4182	TTGTAAAACGACGGCCAGTGCCACGATGGCTACTGCACCACCCAAAT	*gadDT* knockout
WZ4183	AGCTCATGTGTGGTTCCTCCTGTGAGGTGAGATACA	
WZ4184	GGAGGAACCACACATGAGCTGTCCGGTGAATAACCCGAAGGAA	
WZ4185	CGAGCTCGGTACCCGGGGATCCAATGGGTGGAACACGATCAGGT	
WZ4186	TTGTAAAACGACGGCCAGTGCCACGATGGCTACTGCACCACCCAA	*potE* integration
WZ4187	GGTATTTATGTCAACACCGCCAGTTTTTTCCTTCGGGTTATTCACCGGA	
WZ4188	TGGCGGTGTTGACATAAATACCACT	
WZ4189	TTAACCGTGTTTATTTTTCAGTTCAAAGCGT	
WZ4190	ACGCTTTGAACTGAAAAATAAACACGGTTAATCACTTCCTGTTGTGGCTGCCT	
WZ4191	AATTCGAGCTCGGTACCCGGGGATCCCTCTTCTAGCGCTTCACCACCAG	

Primers	Sequence (5'-3')	Note
WZ4192	ATGCCTGCAGGTCGACTCTAGAAAAGGAGGAAACTTATGAAAC GTCCGCTGGAAGGTA	*act* amplification to construct pXMJ19-P_{tuf}-*guaB*-*gadM*-*act*
WZ4193	TCCGCCAAAACAGCCAAGCTGAATTCGAGCTCTTAGATAACGTT TTTCTCTTCCA	
WZ867	CGTACTGCTGAAGGCTCTT	RT-PCR for *rpoB* gene
WZ868	TTTGCTACACCATCGGACT	
WZ981	ACCTACACCGACGACGCTGTTTCCG	RT-PCR for *acn* gene
WZ982	GTTGTCAGCTTCGACGCCGCCTTCA	
WZ983	TTCGTATGATCGGTTCCGCACAGGC	RT-PCR for *gltA* gene
WZ984	GTCGCCACCGTGGTTGCTCTTGATG	
WZ985	ACCGTTATCGAAGACTGCCGCAAGA	RT-PCR for *icd* gene
WZ986	TGAACCACACCGTCTGCTTCGATGC	
WZ987	CCAAAGCCAACCCAGGCAGAGCAGA	RT-PCR for *kgd* gene
WZ988	GCGGAGTCCATCAGTGGGATAAGTG	
WZ989	TATCCTGCTGGTCGCATTGGTTCTG	RT-PCR for *sucCD* gene
WZ990	AAGGAGTTGAAGCCGCCAACGAGGT	
WZ991	CAGGCTTCCACTCCCTCAACTACGG	RT-PCR for *aceA* gene
WZ992	AGCTGCCTTGAACTCACGGTTCTGC	
WZ993	CGCAACATTCTCACCATTCCAACCG	RT-PCR for *aceB* gene
WZ994	CACCGTGCTCAACCCAGCGCACAAC	

References

1. Zhang, G.; Wang, W.; Deng, A.; Sun, Z.; Zhang, Y.; Liang, Y.; Che, Y.; Wen, T., A mimicking-of-DNA-methylation-patterns pipeline for overcoming the restriction barrier of bacteria. *PLoS Genet* **2012,** 8, (9), e1002987.
2. Schafer, A.; Tauch, A.; Jager, W.; Kalinowski, J.; Thierbach, G.; Puhler, A., Small mobilizable multi-purpose cloning vectors derived from the *Escherichia coli* plasmids pK18 and pK19: selection of defined deletions in the chromosome of *Corynebacterium glutamicum*. *Gene* **1994,** 145, (1), 69-73.
3. Zhang, Y.; Cai, J.; Shang, X.; Wang, B.; Liu, S.; Chai, X.; Tan, T.; Zhang, Y.; Wen, T., A new genome-scale metabolic model of *Corynebacterium glutamicum* and its application. *Biotechnol Biofuels* **2017,** 10, 169.
4. Zhang, Y.; Zhang, Y.; Shang, X.; Wang, B.; Hu, Q.; Liu, S.; Wen, T., Reconstruction of tricarboxylic acid cycle in *Corynebacterium glutamicum* with a genome-scale metabolic network model for trans-4-hydroxyproline production. *Biotechnol Bioeng* **2019,** 116, (1), 99-109.
5. Jakoby, M.; Ngouoto-Nkili, C.; Burkovski, A., Construction and application of new *Corynebacterium glutamicum* vectors. *Biotechnology Techniques* **1999,** 13, (6), 437-441.
